# Benign prostatic hyperplasia is a significant risk factor for bladder cancer in diabetic patients: a population-based cohort study using the National Health Insurance in Taiwan

**DOI:** 10.1186/1471-2407-13-7

**Published:** 2013-01-04

**Authors:** Chin-Hsiao Tseng

**Affiliations:** 1 Department of Internal Medicine, National Taiwan University College of Medicine, Taipei, Taiwan; 2 Department of Internal Medicine, Division of Endocrinology and Metabolism, National Taiwan University Hospital, Taipei, Taiwan; 3 Division of Environmental Health and Occupational Medicine of the National Health Research Institutes, Taipei, Taiwan

**Keywords:** Benign prostatic hyperplasia, Bladder cancer, Diabetes mellitus, Risk factor, Taiwan

## Abstract

**Background:**

Diabetic patients have a higher risk of bladder cancer and benign prostatic hyperplasia (BPH). Theoretically, BPH patients may have an increased risk of bladder cancer because residual urine in the bladder surely increases the contact time between urinary excreted carcinogens and the urothelium. However, whether BPH increases bladder cancer risk in patients with type 2 diabetes has not been studied.

**Methods:**

The reimbursement databases of all Taiwanese diabetic patients under oral anti-diabetic agents or insulin from 1996 to 2009 were retrieved from the National Health Insurance. An entry date was set at 1 January 2006 and a total of 547584 men with type 2 diabetes were followed up for bladder cancer incidence until the end of 2009. Incidences of bladder cancer for BPH by status and by duration were calculated and adjusted hazard ratios (95% confidence intervals) were estimated by Cox regression. The effects of diabetes duration and medications used for diabetic control in relation with bladder cancer risk were also evaluated by Cox regression in BPH men.

**Results:**

The incidences were 258.77 and 69.34 per 100,000 person-years for patients with and without BPH, respectively, adjusted hazard ratio 1.794 (1.572, 2.047). For BPH patients, those who underwent surgical procedures for BPH had a higher incidence than those who did not (355.45 vs. 250.09 per 100,000 person-years), respective adjusted hazard ratios: 2.459 (1.946, 3.109) and 1.709 (1.492, 1.958). The significantly higher risk could be demonstrated for BPH of any duration: respective adjusted hazard ratios 1.750 (1.430, 1.605), 1.844 (1.543, 2.203), 2.011 (1.680, 2.406) and 1.605 (1.341, 1.921) for BPH <1, 1–3, 3–5 and ≥5 years versus patients without BPH. Sensitivity analyses for patients aged ≥60 years and after excluding BPH patients with surgical procedures or without surgical procedures, respectively, yielded similar results. In BPH men, diabetes duration was not significantly related with bladder cancer; but metformin was consistently associated with a significantly lower risk, with adjusted hazard ratio of 0.719 (0.590, 0.875) for all ages and 0.742 (0.604, 0.912) for age ≥60 years.

**Conclusions:**

BPH is a significant risk factor for bladder cancer in men with type 2 diabetes. Metformin may protect against bladder cancer in BPH men.

## Background

Benign prostatic hyperplasia (BPH) is a common urological disorder in men. Its prevalence increases with age and may affect 3 of 4 men in their sixties [1-3]. BPH may cause gradual obstruction to the bladder outflow, leading to progressive severity in lower urinary tract symptoms such as frequency, urgency, nocturia, incomplete voiding and weak urinary stream.

Residual urine in the bladder in patients with BPH surely increases the contact time between urinary excreted carcinogens and the urothelium. Therefore, theoretically, patients with BPH may have an increased risk of bladder cancer. An animal study conducted in female Wistar rats supported such a hypothesis. Matsumoto et al. found that surgically induced partial bladder outlet obstruction resulted in a greater incidence of bladder cancer induced by the carcinogen n-butyl-n-butanol nitrosamine [4].

Human studies investigating the link between BPH and bladder cancer are still sparse. Two early case–control studies suggested an increased risk [5,6]. A recent cohort study recruiting 79280 Swedish men hospitalized for BPH from 1964 to 1983 and following the patients until 1989 concluded that the overall risk of bladder cancer was not increased with BPH, but patients who underwent transurethral resection of the prostate had a significantly 47% higher risk [7].

On the other hand, diabetes may affect the lower urinary tract symptoms/functions [8-13], and patients with type 2 diabetes may have a higher risk of BPH [3,10,12,14-17]. A study estimated that men with higher fasting glucose (>110 mg/dl vs. 110 mg/dl or less) or with a diagnosis of diabetes may have a significantly 3-fold and 2.3-fold higher risk of BPH, respectively [14]. Actually, type 2 diabetes and BPH share several common risk factors including aging, insulin resistance and obesity [3]. Studies suggested that type 2 diabetes, obesity and metabolic syndrome may all affect the growth of BPH. For example, an earlier study showed that the annual BPH growth rate for patients without and with type 2 diabetes was 0.928 ml/year and 1.385 ml/year, respectively [17]. The Baltimore Longitudinal Study of Aging suggested that prostate volume increased 0.41 ml with each 1-kg/m^2^ increment of body mass index, and there was a 3.5-fold higher risk of BPH comparing a body mass index of ≥35 kg/m^2^ to that of <25 kg/m^2^[14]. A study in Turkish men showed that the growth rate of total prostate in BPH patients with and without metabolic syndrome was 1.0 ml/year and 0.64 ml/year, respectively (P = 0.018) [18]. Therefore, the etiology underlying the development of metabolic syndrome including diabetes and obesity may also lead to the growth of prostate.

The number of diabetic patients has been increasing dramatically all over the world in recent decades [19], and this is especially remarkable in the Asian populations [20]. It is estimated that the global number of diabetic patients will increase from 171 million in 2000 to 366 million in 2030; and most of these patients will be seen in Asian countries, with an estimated number of 75 million in 2000 and 180 million in 2030 [19]. Recently, patients with type 2 diabetes have been shown to carry a higher risk of bladder cancer [21-24] and the use of some anti-diabetic agents (e.g. pioglitazone) might be associated with an increased risk [25-28]. However, whether BPH can be a risk factor for bladder cancer in the diabetic patients has not been studied. An elucidation of the association between BPH and bladder cancer is not only an issue of scientific interest, it is also important for the planning and implementing programs for the prevention of bladder cancer, either by reducing the incidence of diabetes and BPH or by early treatment of these diseases. Therefore, the purpose of the present study was to evaluate, in men with type 2 diabetes, whether BPH could be a risk factor for bladder cancer.

## Methods

Since March 1995 a compulsory and universal system of health insurance (the so-called National Health Insurance, NHI) was implemented in Taiwan. All contracted medical institutes must submit computerized and standard claim documents for reimbursement. More than 99% of citizens are enrolled in the NHI, and >98% of the hospitals nationwide are under contract with the NHI. The average number of annual physician visits in Taiwan is one of the highest around the world, at approximately 15 visits per year per capita in 2009.

The National Health Research Institute is the only institute approved, as per local regulations, for handling the NHI reimbursement databases for academic research. The databases contain detailed records on every visit for each patient, including outpatient visits, emergency department visits and hospital admission. The databases also include principal and secondary diagnostic codes, prescription orders, and claimed expenses. The identification information of the individuals was scrambled for the protection of privacy. Diabetes was coded 250.1-250.9, BPH 600, and bladder cancer 188, based on the *International Classification of Diseases, Ninth Revision, Clinical Modification* (ICD-9-CM).

We first retrieved the databases of all patients who had been diagnosed as having diabetes and under treatment with either oral anti-diabetic agents or insulin during the period of 1996–2009 from the whole nation (*n* = 1789776). The selected entry date was 1 January 2006. After excluding patients who had a diagnosis of diabetes after the year 2006 (*n* = 342351), patients who held a Severe Morbidity Card as having type 1 diabetes (*n* = 7120, in Taiwan, patients with type 1 diabetes were issued a so-called “Severe Morbidity Card” after certified diagnosis and they were waived for much of the co-payments), patients having a diagnosis of bladder cancer before 2006 (*n* = 9555), those who died (*n* = 96320) or withdrew from the NHI (*n* = 12502) before entry date, duplicated identification number (*n* = 106), unclear information on date of birth or sex (*n* = 5122), and diabetic patients without any reimbursement record after the entry date (*n* = 235746), a total of 1094404 patients with a diagnosis of type 2 diabetes and under therapy with oral anti-diabetic agents or insulin were recruited. A further exclusion of the female sex yielded 547584 men with type 2 diabetes for the present study.

All comorbidities and covariates were determined as a status/diagnosis before the entry date. The ICD-9-CM codes for the comorbidities were [21,29,30]: nephropathy 580–589, urinary tract disease 590–599, hypertension 401–405, chronic obstructive pulmonary disease (a surrogate for smoking) 490–496, cerebrovascular disease 430–438, ischemic heart disease 410–414, peripheral arterial disease 250.7, 785.4, 443.81 and 440–448, eye disease 250.5, 362.0, 369, 366.41 and 365.44, dyslipidemia 272.0-272.4, heart failure 398.91, 402.11, 402.91, 404.11, 404.13, 404.91, 404.93 and 428, obesity 278, alcohol-related diagnosis 291, 303, 535.3, 571.0, 571.1, 571.2, 571.3 and 980.0, non-alcohol-related chronic liver disease 570–573, 070 and 571.4 (excluding 571.0, 571.1, 571.2 and 571.3), and cancer other than bladder cancer 140–208 (excluding 188). Medications included rosiglitazone, pioglitazone, sulfonylurea, meglitinide, metformin, acarbose, insulin, statin, fibrate, angiotensin-converting enzyme inhibitor and/or angiotensin receptor blocker, calcium channel blocker, non-steroidal anti-inflammatory drugs, alpha-blockers, 5-alpha reductase inhibitors, clopidogrel, ticlopidine, dipyridamole, cyclophosphamide and diuretics. Baseline characteristics between patients with and without BPH were compared by Chi-square test.

The incidence density of bladder cancer was calculated for BPH by status and by duration of BPH diagnosis (<1 year, 1–3 years, 3–5 years and ≥5 years) in patients of all ages and in patients aged ≥60 years, respectively. Patients with BPH were further categorized into two subgroups: with and without receiving surgical procedures for the treatment of BPH, including transurethral incision of the prostate, transurethral resection of the prostate, suprapubic prostatectomy or retropubic prostatectomy. The numerator for the incidence was the number of patients with incident bladder cancer during the 4-year follow-up from 1 January 2006 until 31 December 2009, and the denominator was the person-years of follow-up.

To compare whether patients with BPH had a higher probability of visits to the urologists or receiving laboratory examinations that might potentially lead to the diagnosis of bladder cancer, the frequencies of these items between patients with and without BPH were analyzed by Chi square test, in all patients and in patients aged ≥60 years, respectively. Laboratory examinations included serum tumor markers (including carcinoembryonic antigen, carbohydrate antigen 19–9, carbohydrate antigen 125 and tissue polypeptide antigen), urine cytology, cystoscopy, urinalysis and bladder ultrasonography. The nuclear matrix protein-22 and the bladder tumor associated antigen tests for screening bladder cancer were not reimbursed by the NHI; and therefore they could not be considered in the analyses.

Cox proportional hazards regression was performed to estimate the hazard ratios for bladder cancer for patients with BPH vs. patients without BPH. The following models were created: 1) BPH vs. no BPH; 2) BPH without surgical procedures, BPH with surgical procedures vs. no BPH; and 3) BPH by duration (<1 year, 1–3 years, 3–5 years and ≥5 years) vs. no BPH. For sensitivity analyses, the Cox models were also created after excluding patients with surgical procedures and without surgical procedures, respectively, from the analyses. All models were created for all ages and age ≥60 years, respectively. Age, diabetes duration, comorbidities, medications and potential detection examinations were all adjusted for in the models.

To further evaluate the possible effects of diabetes duration and medications used for diabetic control on the risk of bladder cancer among the BPH men, additional Cox regression models were created for the diabetic men with BPH for all ages and for age ≥60 years, respectively. Age, comorbidities, medications other than anti-diabetic drugs and potential detection examinations were all adjusted for in these models.

Analyses were conducted using SAS statistical software, version 9.1 (SAS Institute, Cary, NC). *P* < 0.05 was considered statistically significant.

## Results

Table 1 compares the baseline characteristics between patients with BPH and those without. Patients with BPH were older and had longer diabetes duration, higher rates of comorbidities (except obesity and alcohol-related diagnosis), and higher rates of using medications and other cancer.

**Table 1:** Baseline characteristics by benign prostatic hyperplasia

**Variables**	**BPH (−)**		**BPH (+)**		***P value***
	***n***	**%**	***n***	**%**	**(Chi-square test)**
*n* = 547584	465485	85.0	82099	15.0	
Age (years)					
<40	28373	6.1	103	0.1	<0.0001
40-49	88077	18.9	1692	2.1	
50-59	144677	31.1	11014	13.4	
60-69	106959	23.0	22785	27.8	
≥70	97399	20.9	46505	56.7	
Diabetes duration (years)					
<1	43069	9.3	1921	2.3	<0.0001
1-3	83311	17.9	6379	7.8	
3-5	7748	16.7	9061	11.0	
≥5	261623	56.2	64738	78.9	
Nephropathy	53002	11.4	19913	24.3	<0.0001
Urinary tract diseases	59012	12.7	33818	41.2	<0.0001
Hypertension	248296	53.3	63518	77.4	<0.0001
Chronic obstructive pulmonary disease	60418	13.0	25837	31.5	<0.0001
Cerebrovascular disease	59318	12.7	23756	28.9	<0.0001
Ischemic heart disease	95649	20.6	33370	40.7	<0.0001
Peripheral arterial disease	52064	11.2	18708	22.8	<0.0001
Eye disease	75016	16.1	24855	30.3	<0.0001
Dyslipidemia	231613	49.8	42928	52.3	<0.0001
Heart failure	25584	5.5	11400	13.9	<0.0001
Obesity	6360	1.37	779	0.95	<0.0001
Alcohol-related diagnosis	14797	3.18	1885	2.30	<0.0001
Non-alcohol-related chronic liver disease	155679	33.44	30421	37.05	<0.0001
Rosiglitazone	64221	13.8	16898	20.6	<0.0001
Pioglitazone	22241	4.8	5191	6.3	<0.0001
Sulfonylurea	365375	78.5	74154	90.3	<0.0001
Meglitinide	51909	11.2	14783	18.0	<0.0001
Metformin	325652	70.0	69254	84.4	<0.0001
Acarbose	68578	14.7	18395	22.4	<0.0001
Insulin	68122	14.6	20951	25.5	<0.0001
Statin	133169	28.6	26302	32.0	<0.0001
Fibrate	106427	22.9	19705	24.0	<0.0001
Angiotensin-converting enzyme inhibitor/angiotensin receptor blocker	198383	42.6	52076	63.4	<0.0001
Calcium channel blocker	131769	28.3	40288	49.1	<0.0001
Non-steroidal anti-inflammatory drugs	303900	65.3	72262	88.0	<0.0001
Alpha-blockers	50456	10.84	69086	84.15	<0.0001
5-alpha reductase inhibitors	425	0.09	3409	4.15	<0.0001
Clopidogrel	14395	3.09	5346	6.51	<0.0001
Ticlopidine	11315	2.43	5278	6.43	<0.0001
Dipyridamole	78550	16.87	30566	37.23	<0.0001
Cyclophosphamide	456	0.10	147	0.18	<0.0001
Diuretics	103397	22.21	36462	44.41	<0.0001
Other cancer prior to baseline	41311	8.9	18433	22.5	<0.0001

*BPH* benign prostatic hyperplasia.

Table 2 shows the crude incidence of bladder cancer with regards to BPH status or duration. The incidence was 69.34 per 100,000 person-years for patients without BPH and was 258.77 per 100,000 person-years for patients with BPH. For BPH patients, those who underwent a surgical procedure had a higher incidence than those who had not received a surgical procedure (355.45 vs. 250.09 per 100,000 person-years). The incidence seemed to increase with longer duration of BPH and peaked at BPH duration of 3–5 years, especially in those who underwent a surgical procedure. The findings were similar in the analyses for those aged ≥60 years.

**Table 2:** Incidence of bladder cancer with regards to benign prostatic hyperplasia by status or duration with and without surgical procedures

**BPH status/duration**	**Case number**	***n*of Incident bladder cancer**	**%**	**Person-years**	**Incidence rate**
					**(per 100,000 person-years)**
**All ages**					
**I. BPH by status**					
No BPH	462432	1127	0.24	1625241.83	69.34
BPH	85152	742	0.87	286736.17	258.77
BPH/procedures (−)	78526	658	0.84	263103.83	250.09
BPH/procedures (+)	6626	84	1.27	23632.33	355.45
**II. BPH by duration**					
All BPH patients					
<1 year	16163	126	0.78	54808.83	229.89
1-3 years	23955	199	0.83	81308.00	244.75
3-5 years	20649	203	0.98	69799.92	290.83
≥5 years	24385	214	0.88	80819.42	264.79
BPH/procedures (−)					
<1 year	12421	107	0.86	41550.17	257.52
1-3 years	23005	180	0.78	77899.17	231.07
3-5 years	19952	186	0.93	67255.08	276.56
≥5 years	23148	185	0.80	76399.42	242.15
BPH/procedures (+)					
<1 year	3742	19	0.51	13258.67	143.30
1-3 years	950	19	2.00	3408.83	557.38
3-5 years	697	17	2.44	2544.83	668.02
≥5 years	1237	29	2.34	4420.00	656.11
**Age ≥60 years**					
**I. BPH by status**					
No BPH	201704	779	0.39	685559.17	113.63
BPH	71944	684	0.95	238880.17	286.34
BPH/procedures (−)	65952	605	0.92	217633.42	277.99
BPH/procedures (+)	5992	79	1.32	21246.75	371.82
**II. BPH by duration**					
All BPH patients					
<1 year	12864	111	0.86	42903.33	258.72
1-3 years	19405	182	0.94	64903.08	280.42
3-5 years	17536	193	1.10	58425.00	330.34
≥5 years	22139	198	0.89	72648.75	272.54
BPH/procedures (−)					
<1 year	9567	93	0.97	31307.58	297.05
1-3 years	18529	164	0.89	61772.17	265.49
3-5 years	16885	176	1.04	56058.00	313.96
≥5 years	20971	172	0.82	68495.67	251.11
BPH/procedures (+)					
<1 year	3297	18	0.55	11595.75	155.23
1-3 years	876	18	2.05	3130.92	574.91
3-5 years	651	17	2.61	2367.00	718.21
≥5 years	1168	26	2.23	4153.08	626.04

*BPH* benign prostatic hyperplasia, *HR* hazard ratio, *CI* confidence interval.

Table 3 compares the frequency of visits to urologists and laboratory examinations that might potentially lead to the diagnosis of bladder cancer in patients with and without BPH. It is true that patients with BPH had a significantly higher probability of potential detection bias.

**Table 3:** Comparison of the frequency of visits to urologists and laboratory examinations potentially leading to diagnosis of bladder cancer in patients with and without benign prostatic hyperplasia

**Variables**	**Benign prostatic hyperplasia**				***P value***
	**No**		**Yes**		
	**n**	**%**	**n**	**%**	
**All ages**					
Visits to urologists					
No	427531	91.85	59460	72.42	<0.0001
Yes	37954	8.15	22639	27.58	
Laboratory examinations*					
No	119766	25.73	10354	12.61	<0.0001
Yes	345719	74.27	71745	87.39	
Any of the above					
No	116896	25.11	9309	11.34	<0.0001
Yes	348589	74.89	72790	88.66	
**Age ≥60 years**					
Visits to urologists					
No	185836	90.94	50441	72.80	<0.0001
Yes	18522	9.06	18849	27.20	
Laboratory examinations*					
No	47633	23.31	8460	12.21	<0.0001
Yes	156725	76.69	60830	87.79	
Any of the above					
No	46463	22.74	7663	11.06	<0.0001
Yes	157895	77.26	61627	88.94	

*Laboratory examinations include serum tumor markers, urine cytology, cystoscopy, urinalysis and bladder ultrasonography.

The hazard ratios with regards to BPH by status or duration are presented in Table 4. Except for BPH by duration <1 year in the sensitivity analyses after excluding BPH patients without a surgical procedure from the analyses, all hazard ratios showed a significantly higher risk of bladder cancer associated with BPH. For analyses in all patients of all ages, patients with BPH had a significantly higher risk, with hazard ratio (95% confidence interval): 1.794 (1.572, 2.047). BPH patients with a surgical procedure had a larger magnitude of hazard ratio than those without: 2.459 (1.946, 3.109) vs. 1.709 (1.492, 1.958). The significantly higher risk could be demonstrated in patients with BPH of any duration. The analyses were similar in the sensitivity analyses, but the magnitudes of hazard ratios were larger in patients with a surgical procedure than those without a surgical procedure in any specific BPH duration (except for the category with BPH duration <1 year).

**Table 4:** Hazard ratios for bladder cancer with regards to benign prostatic hyperplasia

**BPH**	**All ages**			**Age ≥60 years**		
	**HR**	**95% CI**	***P value***	**HR**	**95% CI**	***P value***
**All patients**						
		N = 547584			N = 273648	
I. BPH by status						
1. BPH/procedures (−) or (+)	1.794	(1.572, 2.047)	<0.0001	1.842	(1.600, 2.121)	<0.0001
2. BPH/procedures (−)	1.709	(1.492, 1.958)	<0.0001	1.753	(1.517, 2.027)	<0.0001
BPH/procedures (+)	2.459	(1.946, 3.109)	<0.0001	2.500	(1.960, 3.189)	<0.0001
II. BPH by duration						
<1 year	1.750	(1.430, 1.605)	<0.0001	1.769	(1.425, 2.195)	<0.0001
1-3 years	1.844	(1.543, 2.203)	<0.0001	1.897	(1.571, 2.291)	<0.0001
3-5 years	2.011	(1.680, 2.406)	<0.0001	2.125	(1.760, 2.566)	<0.0001
≥5 years	1.605	(1.341, 1.921)	<0.0001	1.623	(1.343, 1.962)	<0.0001
**Sensitivity analyses**						
**Excluding BPH with surgical procedure**						
		N = 540958			N = 267656	
I. BPH by status	1.740	(1.515, 1.740)	<0.0001	1.797	(1.549, 2.084)	<0.0001
II. BPH by duration						
<1 year	1.943	(1.557, 2.425)	<0.0001	1.975	(1.556, 2.507)	<0.0001
1-3 years	1.756	(1.459, 2.112)	<0.0001	1.813	(1.490, 2.207)	<0.0001
3-5 years	1.913	(1.589, 2.304)	<0.0001	2.025	(1.666, 2.462)	<0.0001
≥5 years	1.474	(1.220, 1.780)	<0.0001	1.506	(1.233, 1.839)	<0.0001
**Excluding BPH without surgical procedure**						
		N = 469058			N = 207696	
I. BPH by status	2.525	(1.984, 3.212)	<0.0001	2.671	(2.078, 3.433)	<0.0001
II. BPH by duration						
<1 year	1.200	(0.760, 1.896)	0.4342	1.275	(0.796, 2.041)	0.3123
1-3 years	3.983	(2.477, 6.404)	<0.0001	4.274	(2.614, 6.988)	<0.0001
3-5 years	4.648	(2.818, 7.668)	<0.0001	5.246	(3.163, 8.701)	<0.0001
≥5 years	3.837	(2.588, 5.689)	<0.0001	3.984	(2.621, 6.057)	<0.0001

*BPH* benign prostatic hyperplasia, *HR* hazard ratio, *CI* confidence interval.

Models are adjusted for all variables in Table 1 and for potential detection examinations in Table 3.

The referent group in the models is patients without BPH.

Table 5 shows the adjusted hazard ratios for bladder cancer with regards to diabetes duration and medications used for diabetic control in the diabetic men with BPH. It was noted that diabetes duration was not significantly related to bladder cancer in the BPH men; and that among all anti-diabetic drugs only metformin was significantly associated with a lower risk of bladder cancer in the BPH men.

**Table 5:** Hazard ratios for bladder cancer with regards to diabetes duration and medications used for diabetic control in diabetic men with benign prostatic hyperplasia

	**All ages**			**Age ≥60 years**		
	**HR**	**95% CI**	***P value***	**HR**	**95% CI**	***P value***
Diabetes duration						
1-3 years vs. <1 year	0.992	(0.637, 1.546)	0.9731	1.117	(0.680, 1.835)	0.6610
3-5 years vs. <1 year	0.735	(0.471, 1.147)	0.1748	0.893	(0.546, 1.461)	0.6534
≥5 years vs. <1 year	0.679	(0.449, 1.027)	0.0664	0.784	(0.494, 1.246)	0.3035
Medications for diabetic control						
Rosiglitazone (Yes vs. No)	1.124	(0.923, 1.369)	0.2443	1.115	(0.908, 1.370)	0.2994
Pioglitazone (Yes vs. No)	1.023	(0.752, 1.393)	0.8846	1.066	(0.774, 1.467)	0.6959
Sulfonylurea (Yes vs. No)	0.939	(0.742, 1.190)	0.6035	0.940	(0.734, 1.205)	0.6252
Meglitinide (Yes vs. No)	1.133	(0.934, 1.375)	0.2057	1.170	(0.958, 1.428)	0.1239
Metformin (Yes vs. No)	0.719	(0.590, 0.875)	0.0010	0.742	(0.604, 0.912)	0.0046
Acarbose (Yes vs. No)	1.045	(0.866, 1.261)	0.6471	1.034	(0.850, 1.258)	0.7407
Insulin (Yes vs. No)	0.893	(0.738, 1.081)	0.3465	0.880	(0.721, 1.073)	0.2062

*HR* hazard ratio, *CI* confidence interval.

Models are adjusted for all variables in Table 1 and for potential detection examinations in Table 3.

## Discussion

The present study strongly suggested that BPH is an important risk factor for bladder cancer in patients with type 2 diabetes (Tables 2 and 4). Furthermore, BPH patients who had received a surgical procedure might have a higher magnitude of hazard ratio than those without a surgical procedure (Tables 2 and 4), suggesting that patients with more severe clinical conditions of BPH might have a higher risk of bladder cancer. Although BPH patients were older, had higher prevalences of most comorbidities and medications used (Table 1) and a higher probability of detection bias (Table 3), the risk associated with BPH could not be ascribed to these confounders (Table 4). The effect of BPH on bladder cancer risk seemed to be independent of diabetes because none of the diabetes duration was significantly related with bladder cancer in the BPH men (Table 5). It was also observed that, in the BPH men, those who used metformin might have a significantly lower risk of bladder cancer than those who had never used metformin (Table 5), suggesting a potentially preventive effect of metformin against bladder cancer development.

A possible explanation for a link between BPH and bladder cancer is that the residual urine in the bladder in patients with BPH may increase the time of urothelial exposure to urinary excreted carcinogens. Such a hypothesis is strongly supported by the animal study by Matsumoto et al. because bladder cancer induced by the carcinogen n-butyl-n-butanol nitrosamine may also be aggravated by surgically induced bladder outlet obstruction in female Wistar rats [4]. Another circumstantial evidence supporting the hypothesis in humans came from prospective [31] and case–control [32] studies showing that high fluid intake, indicating less concentrated urine or more frequent micturition, was associated with a lower risk of bladder cancer.

Patients with BPH also have a significantly higher risk of chronic kidney disease, probably due to an obstructive uropathy [33]. Chronic kidney disease has been consistently proved to be a significant risk factor for bladder cancer in the Taiwanese population [21,34]. Therefore, the increased risk of bladder cancer in patients with BPH could also be partially explained by the higher incidence of chronic kidney disease in these patients.

In the Swedish prospective follow-up study, there was a lack of an overall risk of bladder cancer associated with BPH [7], in contrary to a significant association in the present study (Tables 2 and 4). There were several possible explanations. First, the Swedish study used the bladder cancer incidence in the general population as referents for the calculation of the standardized incidence ratio. The referent groups would surely include the BPH patients in the study and also include other high risk patients like the diabetic patients who may have an increased risk of bladder cancer [21-24]. Second, the Swedish study could not consider the adjustment for potential confounders except for age. Furthermore, different ethnicities might also be a possible explanation. It should be pointed out that the Swedish study did show a significantly 2-fold higher risk of bladder cancer in a subgroup of the BPH men who underwent transurethral resection of the prostate and had genitourinary conditions such as urinary tract infection and stones [7]. This was consistent with the findings of the present study (Tables 2 and 4), and with our previous study showing a significantly higher risk of bladder cancer in patients with a history of urinary tract infection or stones [21].

Although the etiology of BPH remains unknown, metabolic disturbances may promote the growth of prostate. Obesity, insulin resistance with elevated insulin level, and higher serum concentration of insulin-like growth factor-I are associated with a significantly higher risk of BPH [3,35,36]. Obesity and diabetes are also associated with systemic inflammation and oxidative stress, which may promote the inflammatory processes in the prostate, leading to clinical development of BPH [3]. The link between BPH and some lifestyle risk factors of type 2 diabetes such as physical inactivity and dietary factors including increased total energy intake and less vegetables or vitamin D [3,15] also strongly support the existence of some common underlying etiology for BPH and type 2 diabetes, which may well explain the observed association between BPH and type 2 diabetes.

Diabetic patients have an increased risk of bladder cancer [21-24]. The etiology for such a link has not yet been clarified. Some possible mechanisms have been proposed based on elevated blood glucose, insulin resistance, elevated levels of insulin and insulin-like growth factor-I, higher prevalences of neurogenic bladder and sympathetic nerve dysfunction in the diabetic patients [12]. Some recent studies also suggested a possible higher risk of bladder cancer associated with the use of pioglitazone in patients with type 2 diabetes [25-28]. The present study suggested another possible mechanism, which acts through BPH in the diabetic patients.

There are several important clinical implications from the present study. First, BPH-related bladder cancer can be preventable if BPH is prevented or adequately treated at its early stage. Second, in diabetic men with BPH, the use of metformin is potentially preventive for the development of bladder cancer. This finding surely provides a good rationale for the conduction of clinical trials evaluating the use of metformin for the prevention of bladder cancer among high-risk patients with BPH. Third, type 2 diabetes is increasing more remarkably in men than in women in the Taiwanese younger generation in recent 2 decades, probably due to the increasing epidemic of obesity in the young men [37,38]. It is expected that the incidence of bladder cancer in the male population will be increasing in the future when these young and obese diabetic patients become older, because of the link between diabetes and BPH and the higher risk of bladder cancer related to BPH as shown in the present study. Therefore lifestyle modification to reduce the incidence of obesity, diabetes and metabolic syndrome is mandatory not only for preventing the development of BPH, but also for reducing the incidence of bladder cancer. Fourth, the potential confounding effect of BPH should not be neglected while evaluating the association between some medications and bladder cancer risk. For examples, most recent studies evaluating the risk of bladder cancer related to pioglitazone have not seriously considered such a potential confounding from BPH and therefore their conclusion of a positive link could be challenged with a lack of such an adjustment for BPH. Furthermore, an increased risk of bladder cancer has been warned for a new class of oral anti-diabetic agents being under consideration for approval for human use, i.e., the sodium-dependent glucose cotransporter 2 inhibitors with a novel mechanism of inhibiting glucose reabsorption from the kidney [39]. It is strongly suggested that the potential confounding effect of BPH on bladder cancer risk should be assessed during the evaluation for its approval.

Because all diabetic patients were treated with oral anti-diabetic medications or insulin at entry, the chance of misclassification of diabetes was actually very low. However, we could not exclude the possibility of misclassification of bladder cancer and BPH. The probability of misclassification of bladder cancer must be low, because labeled diagnoses should be printed out in all prescriptions handed to the patients. Mislabeling of a cancer diagnosis would not be acceptable to the patients when they saw the diagnosis. With regards to the misclassification of BPH, this might not occur in patients who had received a surgical procedure. Our analyses in different subgroups of the BPH patients (Tables 2 and 4) did not favor a possible impact resulting from such a misclassification. It should be stressed that underdiagnosis of BPH was possible, especially when it was in the early stage without remarkable symptoms. However, if BPH increased the risk of bladder cancer, a misclassification of patients with BPH in the referent group would only have underestimated the hazard ratios.

Because the databases were derived from the whole population and they spanned the whole period from the beginning of the NHI to the end of 2009, there was no concern of potential selection bias related to sampling error. The longitudinal nature of the study and the use of medical records reduced the likelihood of reverse causality and recall bias. However, it should also be mentioned that the local law restricts to retrieve only a certain percentage of the reimbursement data from the whole NHI databases for academic research. Therefore, it was not possible for the present study to include the detailed information on surgical procedures for bladder cancer and additional data from non-diabetic general population for more in-depth analyses on the use of total cystectomy as a surrogate for “invasive bladder cancer” and for a comparison of bladder cancer risk associated with BPH in non-diabetic Taiwanese men, respectively.

Smoking is an important risk factor for bladder cancer [40], but we did not have information of smoking for adjustment and could only consider surrogates that are highly related to smoking, such as chronic obstructive pulmonary disease, ischemic heart disease, cerebrovascular disease and peripheral arterial disease (Table 4). Theoretically, a confounder should be correlated simultaneously with both the exposure (BPH) and the outcome (bladder cancer), and it should not be an intermediate between exposure and outcome [41]. There is no evidence showing smoking as a determinant for BPH [2,15,16].

This study has several strengths. The databases included all claim records on outpatient visits, emergency department visits and hospital admission, and we caught the diagnoses from all sources. Cancer is considered a severe morbidity by the NHI and most medical co-payments can be waived. Furthermore, there is a low drug cost-sharing required by the NHI and patients with certain conditions such as low-income household, veterans or patients with prescription refills for chronic disease are exempted from the drug cost-sharing [42]. Therefore the detection rate of bladder cancer would not tend to differ among different social classes.

The study limitations included a lack of actual measurement data for confounders such as obesity, smoking, alcohol drinking, water intake, family history, lifestyle, diet, hair dye use, and some occupational exposure and genetic parameters. In addition, we did not have biochemical data for evaluating their impact. Another limitation is the lack of information on the grading and staging of bladder cancer.

## Conclusions

This population-based cohort study in Taiwan suggests that BPH is a significant risk factor for bladder cancer in men with type 2 diabetes. Metformin may protect against bladder cancer in the BPH men. This commonly seen risk factor of BPH should not be neglected in future studies evaluating bladder cancer risk.

## Abbreviations

BPH: Benign prostatic hyperplasia; ICD-9-CM: International Classification of Diseases, Ninth Revision, Clinical Modification; NHI: National Health Insurance.

## Competing interests

No competing interests to declare.

## Authors’ contributions

CH researched data and wrote manuscript.

## Pre-publication history

The pre-publication history for this paper can be accessed here:

http://www.biomedcentral.com/1471-2407/13/7/prepub
